# Skeletal and dental effects produced by functional regulator-2 in pre-pubertal class II patients: a controlled study

**DOI:** 10.1186/2196-1042-14-18

**Published:** 2013-07-26

**Authors:** Letizia Perillo, Alberta Femiano, Stefano Palumbo, Luca Contardo, Giuseppe Perinetti

**Affiliations:** Dipartimento di Discipline Odontostomatologiche, Ortodontiche e Chirurgiche, Via Luigi De Crecchio 6, Naples, 80138 Italy; Department of Medical, Surgical and Health Sciences, Ospedale Maggiore, University of Trieste, Piazza Ospitale 1, Trieste, 34129 Italy

## Abstract

**Background:**

Whether skeletal effects are obtained by functional appliances in class II subjects is still controversial. In this regard, most of the available studies did not clearly identify the growth phases (i.e. pubertal or not) of the treated patients. This retrospective controlled study aimed at evaluating the skeletal and dental changes in class II subjects produced by the functional regulator (FR)-2 treatment during the pre-pubertal growth phase.

**Methods:**

The data were derived from records obtained at a university dental clinic. A total of 17 treated subjects and a total of 17 untreated controls, all pre-pubertal, matched for malocclusion, age (8.8 ± 1.5 years) and sex (18 females, 16 males), were included. The overall observational period was 1.6 ± 0.8 years for both groups.

**Results:**

Only minor skeletal changes with very little clinical relevance were seen after the observational period. Most of the changes produced by the FR-2 treatment were at the dental level including palatal tipping of the maxillary incisors and slight proclination of the mandibular incisors, both accounting for the noteworthy overjet reduction.

**Conclusions:**

The present study has shown that functional treatment of class II malocclusion by FR-2 appliance during the pre-pubertal growth phase is limited to modification at the dental level.

## Background

class II is one of the most prevalent dental and skeletal malocclusion in the sagittal plane, and it occurs in up to one-third of the population [1, 2], with the highest prevalence among northern European descent [1]. Although variable combinations of dental and skeletal factors contribute to this malocclusion, the most frequent diagnostic finding in class II malocclusion is mandibular skeletal retrusion [1, 3]. A therapy able to enhance mandibular growth is thus indicated in class II patients [4]. To this goal, a wide range of functional appliances aimed to stimulate mandibular growth by forward posturing of the mandible is available [5]. Many treatment protocols, sample sizes and research approaches have led to disparate outcomes in studies on human subjects [5].

One of the very earlier available functional appliances is the functional regulator (FR)-2 [6]. The FR-2 has been developed to eliminate functional disorders that can interfere with normal skeletal and dental development [6]. The claimed mechanism of action of this appliance is targeting poor postural behaviour of the orofacial musculature and, in particular for class II malocclusion, advancing the mandible with muscular training [6].

In spite of the number of studies on FR-2 treatment performed to date, there are still controversial aspects mainly regarding the skeletal effects provided by this appliance [7]. In particular, a wide range of different results has been reported, from no skeletal effects [8] to restriction of the maxillary growth [9] or enhanced mandibular length [3, 10, 11]. However, major limitations of most of these previous investigations, which may account for the different conclusions carried out, have recently been uncovered [7].

An important aspect in functional treatment for skeletal class II malocclusion is the growth phase, i.e. pre-pubertal or pubertal, at which treatment is delivered. Indeed, it has been demonstrated how individual responsiveness to treatment, especially in terms of mandible elongation, is critically dependent on this aspect [12–14]. However, studies on the FR-2 treatment have included patients on the basis of dentition phase [3] or chronological age [8–10, 15].

Moreover, the request for early treatment in class II patients has led to a common practice procedure according to which functional treatment is performed during a pre-pubertal growth phase. Therefore, considering important clinical implications, the knowledge on whether or not the FR-2 treatment may be effective in reducing class II malocclusion by relevant skeletal changes when delivered at the pre-pubertal growth phase would be useful. The present controlled study was thus carried out to elucidate on the skeletal and dental effects produced by a FR-2 treatment in subjects with skeletal class II malocclusion and treated during the pre-pubertal growth phase.

## Methods

### Subjects and study design

This study followed a retrospective, longitudinal, single-blind design. An initial sample of 128 subjects seeking orthodontic treatment, who had never been treated before and presenting at the Section of Orthodontics of the Department of Oral Sciences, Second University of Naples, was screened. As a routine procedure, a signed informed consent to release diagnostic records for scientific purposes was obtained from the parents of the subjects prior to entry into the treatment. Other inclusion criteria were as follows: (1) skeletal class II malocclusion by mandibular retrusion, (2) good general health with no growth or nutritional problems, (3) European (white) ancestry, (4) absence of major craniofacial or dental anomalies and (5) availability of pre-treatment and post-treatment records all during the pre-pubertal growth phase.

Class II division 1 malocclusion was strictly diagnosed at baseline according to the following signs [10, 16, 17]: full- or half-cusp class II molar relationship, excessive overjet (>4 mm), skeletal sagittal relationship of class II (ANB angle > 4°), mandibular retrusion (SNB angle < 78°) and no maxillary protrusion (SNA angle > 84°). Subjects who refused to be treated at the initial visit but re-presented later were included in the control group whenever a second set of diagnostic recording was available. After this selection, 34 subjects (18 females and 16 males, mean age 8.8 ± 1.5 years), 17 treated and 17 untreated controls, were included in the study.

### Observational term and treatment

Lateral cephalograms were taken at two time periods referred to as T0 (baseline) and T1 (end of observational term). At T0, the mean ages were 8.9 ± 1.1 years for the treated group and 8.9 ± 1.8 years for the untreated control group. At T1, the mean ages were 10.4 ± 1.1 years for the treated group and 10.5 ± 2.0 years for the untreated control group. The treatment/observational period was 1.6 ± 0.8 years for both groups.

The FR-2 appliances were constructed according to the design recommended by Fränkel and Fränkel [6] with an initial mandibular advancement that did not exceed about 3 mm, followed by subsequent step-by-step advancement of the same entities. After FR-2 treatment, care was taken to ensure that the mandible could not be retruded clinically, e.g. dual bite. Treatment was interrupted when a class I molar relationship was achieved.

To discriminate between the lack of effect due to the treatment protocol and poor patients' compliance, only those who declared to have worn the appliance for at least 18 h a day during the first 12 months of the treatment were selected herein [10]. This judgment was based on routine reports from parents of the patients. The initial lateral cephalogram was obtained not earlier than 4 months before the onset of the FR-2 treatment, and the final one was obtained not later than 2 months after the end of treatment.

### Assessment of the pre-pubertal growth phase

Pre-pubertal growth phase was assessed through the third middle phalanx maturation (MPM) method [18]. The MPM method as proposed herein comprises five stages (MPS), of which stages 1 and 2 are present in the pre-pubertal subjects and were briefly defined as follows:

*MPS1* is when the epiphysis is narrower than the metaphysis or when the epiphysis is as wide as the metaphysis but with both tapered and rounded lateral borders. The epiphysis and metaphysis are not fused. This stage was described to be attained more than 1 year before the onset of the pubertal growth spurt [13].

*MPS2* is when the epiphysis is at least as wide as the metaphysis with sides of increasing thickness and showing a clear line of demarcation at right angle. In case of asymmetry between the two sides, e.g. one typical of MPS2 and the other less mature, the former is used to assign the stage. This stage was described to be attained 1 year before the onset of the pubertal growth phase [13, 19].

All the included subjects had to show an MPS1 or MPS2 at T1.

### Cephalometric analysis

**Figure 1:**
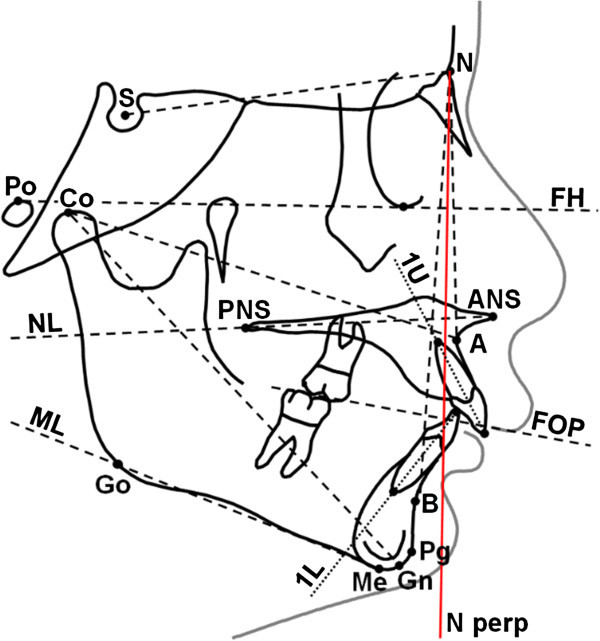
**Landmarks, distances and planes used in the chephalometric analysis.** Planes of reference: FH, Frankfurt horizontal plane; NL, nasal line; ML, mandibular line; FOP, functional occlusal plane; N perp, line on N perpendicular to the FH.

A customised digitization regimen and analysis with cephalometric software (Viewbox, version 3.0, dHAL Software, Kifissia, Greece) was used for all cephalograms examined in this study. The cephalometric analysis required the digitization of 17 landmarks. The customised cephalometric analysis included measurements from the analyses of Steiner [20], Jacobson [21] and McNamara [22], generating 17 variables, eight angular and nine linear, for each tracing (Figure 1). Lateral cephalograms of both treated and untreated subjects at T0 and T1 were standardised as to magnification factor (8%).

All sets of cephalograms were traced at the same time by a blind operator. A preliminary tracing was made for each film in the series, with particular attention to tracing the outlines of the maxilla and the mandible, including the mandibular condyle. A second blind investigator checked each tracing for accuracy. Individual changes were expressed as total, i.e. not annualised.

### Sample size calculation and method error analysis

A sample size of at least 17 subjects *per* group was necessary to detect an effect size (ES) coefficient [23] of 1.0 for each cephalometric parameter in the comparison between the groups at T1, with an alpha set at 0.05 and a power of 0.8 [24]. The ES coefficient is the ratio of the difference between the recordings of the two groups, divided by the within-subject standard deviation (SD). In particular, the ES coefficient has been defined as the ratio of the difference between the mean changes of the two groups divided by the corresponding weighted SDs. A threshold of 0.8 [23] or 1.0 [25] has been reported to be indicative of a clinically relevant effect.

With the aim of quantifying the full method error of the recordings for each cephalometric parameter, the method of moments variance estimator was used [26, 27]. This variance estimator has the advantages of not being affected by any unknown bias, i.e. systematic errors, between pairs of measurements [27]. This analysis was performed on 20 pairs of recordings randomly selected.

### Statistical analysis

The SPSS software, version 13.0 (SPSS® Inc., Chicago, IL, USA) and Comprehensive Meta-Analysis, version 2 (BiostatTM, Englewood, NJ, USA) were used to perform the statistical analyses. After having tested the normality of the data with the Shapiro-Wilk test and the equality of variance among the datasets using a Levene test, non-parametric methods were used for data analysis. Nevertheless, data were summarised as mean ± SD. For each of the cephalometric parameters, a Wilcoxon test and a Mann–Whitney *U* test were used to test the significance of the differences between the time points within each group and between the two groups within either time point, respectively.

For both treated and untreated control groups, the total changes for all cephalometric variables between T1 and T0 were computed. The significance of the differences in these changes between the groups was also evaluated by the Mann–Whitney *U* test. Finally, the changes seen in the treated group subtracted by the corresponding ones seen in the untreated control group were referred to as the ‘treatment effect’, and for these, the ES coefficients along with the 95% confidence intervals (CIs) were calculated, as previously described [28].

Briefly, the ES coefficients have been calculated to assess the results in terms of statistically and/or clinically significant differences. In particular, the ES coefficient has been defined as the ratio of the difference between the mean changes of the two groups divided by the corresponding weighted SDs. Even though a threshold of 0.2 [23] has been reported to be indicative of the minimal value to assess the existence of a ‘small effect’, herein the null hypothesis for the ES coefficient was to be equal to zero, i.e. when the 95% CI includes the zero value, then ‘no treatment effect’ was assessed. A *p* <0.05 was used to reject the null hypothesis.

## Results

The errors for linear measurements ranged from 0.4 (overjet) to 0.9 mm (Pg to N perp); the errors for angular measurements varied from 0.4° (ANB angle) to 1.1° (FH/ML angle). All results regarding the cephalometric skeletal parameters are summarised in Table 1. In comparisons between the groups, none of the differences between the values were statistically significant at either T0 or T1, with the exception of the ANB angle recorded at T1 that was significantly lower in the treated group. In comparisons over time, within each group, condylion-A (Co-A), condylion-gnathion (Co-Gn) and maxillo-mandibular length (Max-Mand) difference underwent significant increases in both the treated and untreated control groups (*p* < 0.01, at least). Only for the treated group, the SNB angle underwent a slight (0.5°) but significant (*p* < 0.05) increase, and the ANB angle, Wits and NL/ML angle underwent a significant reduction of 1.7°, 1.4 mm and 0.9°, respectively (*p* < 0.05, at least). Moreover, the changes seen over time in the two groups were not significantly different for any parameter, with the exception of the ANB angle that underwent a greater reduction in the treated group (treatment effect, 1.0°, *p* < 0.05). The ES coefficients for the treatment effects were all not significantly different than zero, except for the ANB angle that was 0.8 (95% CI, 0.1-1.5).

**Table 1:** **Chephalometric skeletal parameters of the groups and corresponding changes (** ***n*** **= 17 per group)**

Parameter	Group	Time point		Changes	ES coefficient
		T0	T1		
Maxillary					
SNA angle (°)	Treated	80.6 ± 2.4	80.2 ± 2.5	−0.4 ± 1.3	0.1 (−0.6-0.7)
	Control	79.8 ± 2.7	79.5 ± 2.8	−0.3 ± 1.5	
Difference		NS	NS	NS	NS
A to N perp (mm)	Treated	1.8 ± 2.5	1.1 ± 2.9	−0.7 ± 1.6	0.2 (−0.5-0.8)
	Control	2.2 ± 2.5	1.8 ± 2.9	−0.4 ± 1.6	
Difference		NS	NS	NS	NS
Co-A (mm)	Treated	85.1 ± 3.8	87.6 ± 4.3*	2.5 ± 1.9	0.2 (−0.4-0.9)
	Control	82.7 ± 3.2	84.7 ± 3.8*	2.0 ± 2.1	
Difference		NS	*p* < 0.05	NS	NS
Mandibular					
SNB angle (°)	Treated	74.2 ± 2.0	74.7 ± 2.3**	0.5 ± 1.0	0.1 (−0.6-0.7)
	Control	73.6 ± 2.2	74.0 ± 2.5	0.4 ± 1.2	
Difference		NS	NS	NS	NS
Pg to N perp (mm)	Treated	−6.9 ± 3.9	−7.3 ± 4.5	−0.4 ± 2.0	0.3 (−0.4-0.9)
	Control	−5.0 ± 3.4	−4.8 ± 4.8	0.2 ± 2.1	
Difference		NS	NS	NS	NS
Co-Gn (mm)	Treated	103.6 ± 4.6	107.7 ± 5.3***	4.1 ± 2.2	0.0 (−0.6-0.7)
	Control	101.9 ± 4.4	105.9 ± 6.2***	4.0 ± 3.0	
Difference		NS	NS	NS	NS
Maxillo-mandibular					
ANB angle (°)	Treated	6.5 ± 1.5	4.8 ± 0.9***	−1.7 ± 1.2	0.8 (−0.1-1.5)
	Control	6.2 ± 1.2	5.5 ± 1.1	−0.7 ± 1.3	
Difference		NS	NS	*p* < 0.05	*p* < 0.05
Wits (mm)	Treated	3.8 ± 1.2	2.4 ± 1.3*	−1.4 ± 1.1	0.1 (−0.6-0.8)
	Control	3.1 ± 0.8	2.0 ± 2.6	−1.2 ± 2.5	
Difference		NS	NS	NS	NS
Max-Mand difference (mm)	Treated	18.4 ± 3.3	20.0 ± 2.9***	1.6 ± 1.4	0.3 (−0.4-0.9)
	Control	19.2 ± 2.7	21.3 ± 4.3*	2.1 ± 2.1	
Difference		NS	NS	NS	NS
Vertical					
FH-palatal plane angle (°)	Treated	−2.9 ± 3.4	−2.3 ± 3.8	0.6 ± 1.2	0.4 (−0.3-1.1)
	Control	−4.6 ± 3.1	−4.5 ± 2.8	0.1 ± 1.3	
Difference		NS	NS	NS	NS
FH-mandibular plane angle (°)	Treated	22.5 ± 4.9	22.2 ± 5.1	−0.3 ± 1.1	0.4 (−0.3-1.1)
	Control	22.2 ± 4.9	22.5 ± 4.6	0.2 ± 1.3	
Difference		NS	NS	NS	NS
Palatal plane-mandibular plane (°)	Treated	25.4 ± 3.1	24.4 ± 3.0**	−0.9 ± 1.3	0.6 (−0.0-1.3)
	Control	26.8 ± 5.3	26.9 ± 4.9	0.1 ± 1.7	
Difference		NS	NS	NS	NS

Data are presented as mean ± SD or mean (95% confidence interval). For the ES coefficient, the null hypothesis is to be equal to zero. NS, difference not statistically significant. Significant differences with the corresponding baseline value: *p < 0.01, **p < 0.05, ***p < 0.001.

All results regarding the cephalometric dental parameters are summarised in Table 2. In comparisons between the groups, none of the differences between the values were statistically significant at T0. On the contrary, at T1, molar relationship and L1/ML angle were significantly greater in the treated group (*p* < 0.001), while the overjet was significantly lower, again, in the treated group (*p* < 0.05). No significant differences were seen for the U1/FH angle and overbite. In comparisons over time, no significant differences were seen for the untreated control group, while in the treated group, the molar relationship and L1/ML angle underwent a significant increase (*p* < 0.01), and the U1/FH angle and overjet underwent a significant decrease (*p* < 0.01, at least). Moreover, the changes seen over time in the two groups were all significantly different for any parameter, with the exception of the L1/ML angle and overbite. In particular, these changes were greater in the treated group for molar relationship and L1/ML angle (treatment effect, 1.8 mm, *p* < 0.01, at least) and lower in the treated group for U1/FH angle and overjet (treatment effect, 5.3° and 2.9 mm, respectively, *p* < 0.01, at least). In particular, the changes seen in the L1/ML angle yielded a treatment effect of 2.8°, although not significantly different between the groups. The ES coefficients for the treatment effects were all significantly different than zero, except that for the overbite. These significant ES coefficients ranged from 0.7 to the 2.1 for the L1/ML angle and overjet, respectively.

**Table 2:** **Chephalometric dental parameters of the groups and corresponding changes (** ***n*** **= 17 per group)**

Parameter	Group	Time point		Changes	ES coefficient
		T0	T1		
Molar relationship (mm)	Treated	−1.3 ± 1.7	0.9 ± 1.5*	2.2 ± 2.2	1.0 (0.3-1.7)
	Control	−1.8 ± 1.7	−1.4 ± 1.7	0.4 ± 1.1	
Difference		NS	*p* < 0.001	*p* < 0.01	*p* < 0.01
U1/FH angle (°)	Treated	116.6 ± 5.7	112.8 ± 5.9*	−3.8 ± 3.7	1.1 (0.4-1.8)
	Control	113.0 ± 9.3	114.6 ± 8.2	1.5 ± 5.3	
Difference		NS	NS	*p* < 0.01	*p* < 0.01
L1/Mandibular plane angle (°)	Treated	98.5 ± 5.7	101.8 ± 5.1*	3.3 ± 3.5	0.7 (0.0-1.3)
	Control	95.4 ± 5.7	95.9 ± 4.8	0.5 ± 4.8	
Difference		NS	*p* < 0.001	NS	*p* < 0.05
Overjet (mm)	Treated	8.3 ± 1.5	5.3 ± 1.4**	−3.1 ± 1.3	2.1 (1.3-2.9)
	Control	7.5 ± 2.1	7.3 ± 2.4	−0.2 ± 1.4	
Difference		NS	*p* < 0.05	*p* < 0.001	*p* < 0.01
Overbite (mm)	Treated	2.8 ± 1.6	3.7 ± 1.5	0.9 ± 2.1	0.2 (−0.5-0.8)
	Control	3.6 ± 1.9	4.2 ± 1.5	0.6 ± 1.4	
Difference		NS	NS	NS	NS

Data are presented as mean ± SD or mean (95% confidence interval). For the ES coefficient, the null hypothesis is to be equal to zero. NS, difference not statistically significant. Significant difference with the corresponding baseline value: *p < 0.01, **p < 0.001.

## Discussion

Through a strict selection of subjects with skeletal class II malocclusion, the present controlled study addressed what the skeletal and dental effects produced by FR-2 treatment delivered at a pre-pubertal growth phase are. The present results show clinically irrelevant skeletal effects and more pronounced dental changes, especially at the incisors, mainly responsible for the reduction of the overjet. Among the incisors, the maxillary ones underwent more relevant positional changes.

One of the most critical aspects in functional treatment of class II patients relates to the possibility of enhancing mandibular growth to a clinically relevant level. Ideally, this growth should be restored to be comparable to that of class I subjects [5, 29]. In spite of the great number of investigations performed over the last decades [5], most of these were significantly hampered by the designs used [30]. Among the main limitations are improper diagnosis of skeletal class II malocclusion [30] and the lack of evaluation of the growth phase during treatment [5, 7, 31]. Unfortunately, studies on the skeletal effects of FR-2 in growing class II patients are within these general limitations and with few exceptions [11, 12], with a previous meta-analysis [7] that could not clearly discriminate between true treatment effects due to FR-2 wearing and those due to other confounding factors, such as the growth phase at which treatment was delivered.

The present study, even though used a retrospective design, followed a rigorous classification of the class II patients and selected a well-matched control group. For both groups, the whole observational term was within the pre-pubertal growth phase. Having the class II patients included herein characterised by a retruded mandible, the main issue to be addressed is whether or not this treatment protocol is able to enhance mandibular growth on the sagittal plane. In this regard, total mandibular length measured by the Co-Gn (or Co-Menton/Pogonion) distance is a primary parameter. However, one of the major confounding problems in cephalometrics is the use of both Co and Articulare (Ar) as the posterior end point in measuring mandibular total length. Indeed, measurements with Ar as an end point, such as Ar-Gn, might give significant values for supplementary mandibular growth, without a corresponding increase in Co-Gn [15]. In spite of this consideration, several of the previous investigations on functional treatment for class II patients used Ar as the posterior mandibular end point when dealing with different class II appliances [5] or specifically regarding the FR-2 [7]. In the present investigation, the total mandibular length, as Co-Gn, underwent very similar mean increases of 4.1 and 4.0 mm in either the treated and control groups, respectively. Similar results with no or minimal effects of FR-2 treatment on mandibular length (as Co-Gn or Co-Pogonion) were seen by previous investigations [15, 32, 33]. However, these studies did not specify which growth phase the patients had during treatment [15, 32, 33] or had an unmatched control group [15]. On the contrary, McNamara et al. [3] reported a supplementary increment of 3.6 mm/2 years in mandibular length (Co-Gn) in patients treated in the late mixed and early permanent dentitions, presumably with several of these patients treated during the pubertal growth phase. Petrovic et al. [12] reported an additional increase in Co-Pogonion ranging from 0.8 to 5.5 mm/year depending on different biological growth categories in subjects treated with the FR-2 at the pubertal growth phase. Freeman et al. [11] reported a total increase in Co-Gn of 3.0 mm in subjects treated by FR-2 as compared to untreated controls over a 10-year-long follow-up. These subjects showed a pre-pubertal and a post-pubertal growth phase at the beginning and end of the treatment, respectively [11].

**Figure 2:**
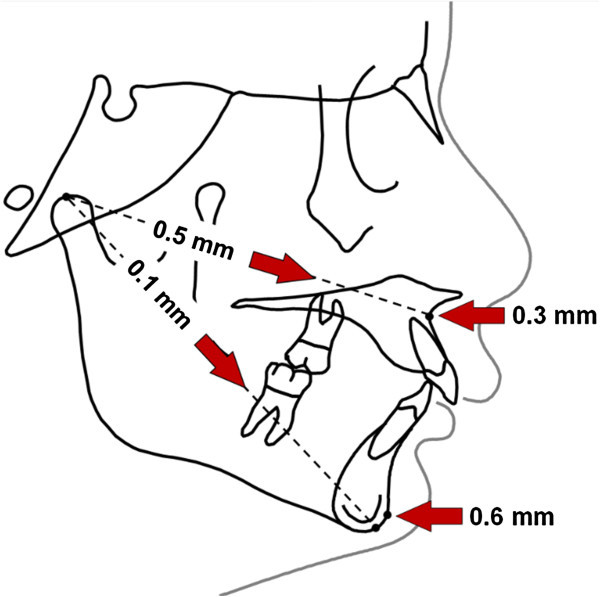
**Treatment effects on main sagittal skeletal parameters.** Treatment effects refer to the difference in mean changes between the time points of the treated and untreated control groups. None of the mean values reported (+0.5 mm (Co-A), +0.1 mm (Co-Gn), −0.3 mm (A to N perp) and +0.6 mm (Pg to N perp)) were in the range of the method error and not statically significant when expressed as effect size coefficient.

In the present study, the treatment effect of FR-2 on the skeletal growth of both the maxilla and mandible was insignificant (Table 1). With the exception of the significant reduction in the ANB angle, all of the other sagittal and vertical parameters showed very similar values between the treated and untreated control groups. Particularly, the ES coefficients were generally low and not significantly different than zero (Table 1). The main treatment effects on the sagittal growth of the maxilla and the mandible are shown in Figure 2. The treatment effects on Co-A and Co-Gn distances were 0.5 and 0.1 mm, respectively. Similarly, the treatment effects on the maxillary and mandibular positions on the sagittal plane, referred to as the A to N perp and Pg to N perp distances, respectively, were also insignificant (+0.3 and +0.6 mm, respectively, Figure 2). These changes were also within the range of the method errors. The previous evidence mentioned above and the present results may thus be explained by the differences in the growth phase at which the patients received treatment. The present controlled study is the only one treating all the patients with FR-2 appliance during the pre-pubertal growth phase. However, pre-pubertal growing patients have been investigated in randomised clinical trials [34, 35] using different functional appliances, although in those studies how the diagnosis of skeletal class II malocclusion was appraised was not clearly reported [36], or they relied on an increased overjet [35] or molar class II (any subdivision) [34] that cannot account for a true skeletal disharmony when used alone [30]. In this regard, the identification of skeletal maturity, with particular regard to the onset of pubertal growth phase, would have major clinical implications when dealing with orthodontic treatment in growing patients with skeletal class II malocclusion [12–14].

Nevertheless, the patients treated herein underwent a noteworthy reduction of the overjet and an improvement of the molar relationship of 2.9 and 1.8 mm, respectively (Table 2). These were, however, due to dental effects produced by the FR-2 treatment. Indeed, a remarkable palatal tipping of the maxillary incisors was seen with a treatment effect on the U1/FH angle of 5.3°. On the other hand, a proclination of the lower incisors was also seen, although only the ES coefficient (0.7), and not the treatment effect on the L1/ML angle (2.8°), was significant. By using different parameters, very similar results have been recorded in previous studies treating patients at the pubertal growth phase [11], late mixed dentition [3] or an unspecified growth/dentition phase [33, 37, 38]. Although Fränkel and Fränkel [6] reported only the insignificant or minimal effects of the FR-2 treatment on the tipping and position of the maxillary and mandibular incisors, all of these evidence would support the concept that FR-2 treatment produced relevant dental effects especially at the incisor level. These dental effects would occur irrespective of the growth phase at which treatment is delivered.

Therefore, considering the lack of skeletal effects by FR-2 treatment and that dental compensation to reduce the overjet may be obtained by a shorter period of fixed appliance therapy, this latter option would be of choice for all pre-pubertal class II subjects with prominent maxillary incisors. This fixed orthodontic treatment, e.g. 2 × 4 appliance, would thus prevent incisor trauma [39], while functional treatment for skeletal growth restoration could be delayed until the onset of the pubertal growth spurt [12–14].

## Conclusions

The FR-2 treatment does not have relevant skeletal effects on class II patients treated during the pre-pubertal growth phase, while the overjet reduction is mainly due to dental effects.

## Authors' information

LP is an associate professor at the Second University of Naples. AF and SP are post-graduate students of the Orthodontic Program at the Second University of Naples. LC is an assistant professor and head of the Orthodontic Program at the University of Trieste. GP is a research fellow at the University of Trieste.
